# Roles of Aquaporins in *Setaria viridis* Stem Development and Sugar Storage

**DOI:** 10.3389/fpls.2016.01815

**Published:** 2016-12-01

**Authors:** Samantha A. McGaughey, Hannah L. Osborn, Lily Chen, Joseph L. Pegler, Stephen D. Tyerman, Robert T. Furbank, Caitlin S. Byrt, Christopher P. L. Grof

**Affiliations:** ^1^Centre for Plant Science, School of Environmental and Life Sciences, University of Newcastle, CallaghanNSW, Australia; ^2^Australian Research Council Centre of Excellence in Plant Energy Biology, Waite Research Institute and School of Agriculture, Food and Wine, University of Adelaide, Glen OsmondSA, Australia; ^3^Australian Research Council Centre of Excellence for Translational Photosynthesis, College of Medicine, Biology and Environment, Australian National University, CanberraACT, Australia

**Keywords:** aquaporin, stem, water transport, sugar accumulation, grasses

## Abstract

*Setaria viridis* is a C_4_ grass used as a model for bioenergy feedstocks. The elongating internodes in developing *S. viridis* stems grow from an intercalary meristem at the base, and progress acropetally toward fully expanded cells that store sugar. During stem development and maturation, water flow is a driver of cell expansion and sugar delivery. As aquaporin proteins are implicated in regulating water flow, we analyzed elongating and mature internode transcriptomes to identify putative aquaporin encoding genes that had particularly high transcript levels during the distinct stages of internode cell expansion and maturation. We observed that *SvPIP2;1* was highly expressed in internode regions undergoing cell expansion, and *SvNIP2;2* was highly expressed in mature sugar accumulating regions. Gene co-expression analysis revealed *SvNIP2;2* expression was highly correlated with the expression of five putative sugar transporters expressed in the *S. viridis* internode. To explore the function of the proteins encoded by *SvPIP2;1* and *SvNIP2;2*, we expressed them in *Xenopus laevis* oocytes and tested their permeability to water. SvPIP2;1 and SvNIP2;2 functioned as water channels in *X. laevis* oocytes and their permeability was gated by pH. Our results indicate that SvPIP2;1 may function as a water channel in developing stems undergoing cell expansion and SvNIP2;2 is a candidate for retrieving water and possibly a yet to be determined solute from mature internodes. Future research will investigate whether changing the function of these proteins influences stem growth and sugar yield in *S. viridis*.

## Introduction

The panicoid grasses sugarcane (*Saccharum officinarum*), sorghum (*Sorghum bicolor*), switchgrass (*Panicum virgatum*), and miscanthus (*Miscanthus X giganteum*) provide the majority of soluble sugars and lignocellulosic biomass used for food and biofuel production worldwide (Somerville et al., 2010; Waclawovsky et al., 2010). A closely related grass with a smaller genome, *Setaria viridis*, is used as a model for these crops in photosynthesis research and for the study of biomass generation and sugar accumulation (Li and Brutnell, 2011; Bennetzen et al., 2012; Brutnell et al., 2015; Martin et al., 2016). The mechanisms that regulate cell expansion and photoassimilate delivery in the stems of these grasses are of interest because they influence the yields of soluble sugars and cell wall biomass produced (Byrt et al., 2011).

Grass stems have repeating units consisting of an internode positioned between two nodes that grow from intercalary meristems at the base; sugar, primarily sucrose, accumulates and is stored in mature cells at the top of the internode (Grof et al., 2013). Along this developmental gradient there is also a transition from synthesis and deposition of primary cell walls through to establishment of thicker secondary cell walls. Sucrose that is not used for growth and maintenance is primarily accumulated intracellularly in the vacuoles of storage parenchyma cells that surround the vasculature (Glasziou and Gayler, 1972; Hoffmann-Thoma et al., 1996; Rae et al., 2005) or in the apoplasm (Tarpley et al., 2007). The mature stems of grasses such as sugarcane can accumulate up to 1M sucrose, with up to 428 mM sucrose stored in the apoplasm (Hawker, 1985; Welbaum and Meinzer, 1990). In addition to a high capacity for soluble sugar storage, carbohydrates are also stored in cell walls of stem parenchyma cells (Botha and Black, 2000; Ermawar et al., 2015; Byrt et al., 2016a).

Historically, increases in sugar yields in the stems of panicoid grasses have been achieved by increasing sugar concentration in stem cells without increasing plant size (McCormick et al., 2009). Sugarcane and sorghum stem sugar content has been increased by years of selecting varieties with the highest culm sucrose content, but these gains have begun to plateau (Grof and Campbell, 2001; Pfeiffer et al., 2010). It may be that we are approaching a physiological ceiling that limits the potential maximum sucrose concentration in the stems of these grasses. Increasing the size of grass stems as a sink may be an effective strategy to increase stem biomass and the potential for greater soluble sugar yield as a relationship exists between stem size and capacity to import and accumulate photoassimilates (sink strength) as soluble sugars or cell wall carbohydrates. Hence, improved stem sugar yields have also been achieved in some sorghum hybrids by expanding stem volume through increased plant height and stem diameter (Pfeiffer et al., 2010; Slewinski, 2012).

In elongating stems, water and dissolved photoassimilates are imported from the phloem into the stem by bulk-flow, or translocation, to drive cell expansion or otherwise be used for growth, development and storage (Schmalstig and Cosgrove, 1990; Wood et al., 1994). In non-expanding storage sinks, water delivering sucrose is likely to be eﬄuxed to the apoplasm and then recycled into the xylem transportation stream to be exported to other tissues (Lang and Thorpe, 1989; Lang, 1990). In addition to vacuolar accumulation of sugars delivered for storage, sugars may also accumulate in the apoplasm with apoplasmic barriers preventing leakage back into the vasculature (Moore, 1995; Patrick, 1997).

The flow of water from the phloem into growth and storage sinks involves the diffusion of water across plant cell membranes facilitated by aquaporins (Kaldenhoff and Fischer, 2006; Zhang et al., 2007). Aquaporins are a highly conserved family of transmembrane channel proteins that enable plants to rapidly and reversibly alter their membrane water permeability or permeability to other solutes depending on the isoform. In maize (*Zea mays*) and rice (*Oryza sativa*) genomes 30–70 aquaporin homologs have been identified, respectively (Chaumont et al., 2001; Sakurai et al., 2005). These large numbers of isoforms can be divided into five sub-families by sequence homology; plasma membrane intrinsic proteins (PIPs), tonoplast intrinsic proteins (TIPs), nodulin-like intrinsic proteins (NIPs), and small basic intrinsic proteins (SIPs; Johanson and Gustavsson, 2002). In dicotyledonous plants but not monocotyledonous plants there is also a group referred to as X intrinsic proteins (XIPs; Danielson and Johanson, 2008).

As aquaporins have important roles in controlling water potential, they are prospective targets for manipulating stem biomass and sugar yields (Maurel, 1997). The crucial role of aquaporins in water delivery to expanding tissues and water recycling in mature tissues is indicated by their high expression in these regions (Barrieu et al., 1998; Chaumont et al., 1998; Wei et al., 2007). Here, we explore the transcriptional regulation of aquaporins in meristematic, expanding, transitional and mature *S. viridis* internodal tissues to identify candidate water channels involved in cell expansion and water recycling after sugar delivery in mature internode tissues.

## Materials and Methods

### Phylogenetic Tree

*Setaria viridis* aquaporins were identified from *S. italica* (Azad et al., 2016), *Arabidopsis* (Johanson et al., 2001), rice (Sakurai et al., 2005), barley (Hove et al., 2015) and maize (Chaumont et al., 2001) aquaporins, and predicted *S. viridis* aquaporins from transcriptomic data (Martin et al., 2016) (Supplementary Table S1) using the online HMMER tool phmmer (Finn et al., 2015^1^). Protein sequences used to generate the phylogenetic tree were obtained for *S. viridis* and *Z. mays* from Phytozome 11.0.5 (*S. viridis* v1.1, DOE-JGI^2^; last accessed July 19, 2016) (Supplementary Table S2). The phylogenetic tree was generated using the neighbor-joining method in the Geneious Tree Builder program (Geneious 9.0.2).

### Elongating Internode Transcriptome Analysis and Aquaporin Candidate Selection

Expression data on identified *S. viridis* aquaporins was obtained from a transcriptome generated from *S. viridis* internode tissue (Martin et al., 2016). Protein sequences of selected putative aquaporin candidates expressed in the elongating *S. viridis* transcriptome were analyzed by HMMscan (Finn et al., 2015^1^).

### Plant Growth Conditions

Seeds of *S. viridis* (Accession-10; A10) were grown in 2 L pots, two plants per pot, in a soil mixture that contained one part coarse river sand, one part perlite, and one part coir peat. The temperatures in the glasshouse, located at the University of Newcastle (Callaghan, NSW, Australia) were 28°C during the day (16 h) and 20°C during the night (8 h). The photoperiod was artificially extended from 5 to 8 am and from 3 to 9 pm by illumination with 400 W metal halide lamps suspended ∼40 cm above the plant canopy. Water levels in pots were maintained with an automatic irrigation system that delivered water to each pot for 2 min once a day. Osmocote^®^ exact slow release fertilizer (Scotts Australia Pty Ltd, Sydney, NSW, Australia) was applied at 20 g per pot, 2 weeks post-germination. Additional fertilization was applied using Wuxal^®^ liquid foliar nutrient and Wuxal^®^ calcium foliar nutrient (AgNova Technologies, Box Hill North, VIC, Australia) alternately each week.

### Harvesting Plant Tissues, RNA Extraction, and cDNA Library Synthesis

Harvesting of plant material from a developing internode followed Martin et al. (2016). Total RNA was isolated from plant material ground with mortar and pestle cooled with liquid nitrogen, using Trizol^®^ Reagent (Thermo Fisher Scientific, Scoresby, VIC, Australia) as per manufacturer’s instruction. Genomic DNA was removed using an Ambion TURBO DNase Kit (Thermo Fisher Scientific) following the manufacturer’s instructions. cDNA was synthesized from 230 ng of isolated RNA from the cell expansion, transitional, and maturing developmental zones as described in Martin et al. (2016) using the Superscript III cDNA synthesis kit (Thermo Fisher Scientific) with an oligo d(T) primer and an extension temperature of 50°C as per the manufacturer’s instructions.

### Reverse-Transcriptase Quantitative PCR (RT-qPCR)

Reverse-transcriptase-qPCR was performed using a Rotor-Gene Q (QIAGEN, Venlo, Netherlands) and GoTaq^®^ Green Master Mix 2x (Promega, Madison, WI, USA). A two-step cycling program was used following the manufacturer’s instructions. The green channel was used for data acquisition. Gene expression of the candidate genes was measured as relative to the housekeeper *S. viridis PP2A* (*SvPP2A*; accession no.: Sevir.2G128000). The *PP2A* gene was selected as a housekeeper gene because it is established as a robust reference gene in many plant species (Czechowski et al., 2005; Klie and Debener, 2011; Bennetzen et al., 2012) and it was consistently expressed across the developmental internode gradient in the transcriptome and cDNA libraries (Martin et al., 2016; Supplementary Figure S1). The forward (F) and reverse (R) primers used for RT-qPCR for were: SvPIP2;1-F (5′-CTCTACATCGTGGCGCAGT-3′) and SvPIP2;1-R (5′–ACGAAGGTGCCGATGATCT-3′), and SvNIP2;2-F (5′–AGTTCACGGGAGCGATGT- 3′) and SvNIP2;2-R (5′–CTAACCCGGCCAACTCAC-3′). SvPIP2;1 and SvNIP2;2 primer sets amplified 161 and 195 base pair fragments from the CDS, respectively. SvPP2A primer set sequences were SvPP2A-F (5′–GGCAACAAGAAGCTCACTCC-3′) and SvPP2A-R (5′-TTGCACATCAATGGAATCGT-3′) and amplified a 164 base pair fragment from the 3′UTR.

### Gene Co-expression Network Analysis

Raw FPKM values of putative aquaporins and sugar transporters were extracted from the *S. viridis* elongating internode transcriptome (Martin et al., 2016). Putative *S. viridis* sugar transporters from the Sucrose Transporter (SUT), Sugar Will Eventually be Exported Transporter (SWEET), and Tonoplast Monosaccharide Transporter (TMT) families were identified by homology to rice SUT, SWEET, and TMT genes (Supplementary Table S3; Supplementary Figures S2–S4). FPKM values were normalized by Log_2_ transformation and Pearson’s correlation coefficients calculated by Metscape (Karnovsky et al., 2012). A gene network was generated for Pearson’s correlation coefficients between 0.8 and 1.0 and visualized with the Metscape app in Cytoscape v3.4.0. Significance of Pearson’s correlation coefficients were calculated using SPSS (IBM Corp. Released 2013. IBM SPSS Statistics for Windows, Version 22.0. Armonk, NY, USA) (Supplementary Table S4). The 1.5Kb 5′ promoter region, directly upstream of the transcriptional start site, of the two aquaporin candidates and the highly correlated putative sugar transporter genes were screened for the presence of *cis*-acting regulatory elements registered through the PlantCARE online database (Lescot et al., 2002^3^) and *cis*-acting elements of *Arabidopsis* and rice SUT genes reported by Ibraheem et al. (2010).

### Photometric Swelling Assay

Extracted consensus coding sequences for *SvPIP2;1* and *SvNIP2;2*, from *S. viridis* transcriptome data (Martin et al., 2016), were synthesized commercially by GenScript (Piscataway, NJ, USA). *SvPIP2;1* and *SvNIP2;2* cDNA fragments were inserted into a gateway enabled pGEMHE vector. pGEMHE constructs were linearized using NheI (New England Biolabs, Ipswich, MA, USA) and purified using the MinElute PCR Purification Kit (QIAGEN). Complimentary RNA (cRNA) for *SvPIP2;1* and *SvNIP2;2* was transcribed using the Ambion mMessage mMachine Kit (Life Technologies, Carlsbad, CA, USA).

*Xenopus laevis* oocytes were injected with 46 ng of *SvPIP2;1* or *SvNIP2;2* cRNA in 46 μL of water, or 46 μL of water alone as a control. Injected oocytes were incubated for 72 h in Ca-Ringer’s solution. Prior to undertaking permeability assays oocytes were transferred into ND96 solution pH 7.4 (96 mM NaCl, 2 mM KCl, 1.8 mM CaCl_2_, 1 mM MgCl_2_, 500 μg.mL^-1^ Streptomycin, 500 μg.mL^-1^ Tetracycline; 204 osmol/L) and allowed to acclimate for 30 min. Oocytes were then individually transferred into a 1:5 dilution of ND96 solution (42 osmol/L), pH 7.4, and swelling was measured for 1 min for *SvPIP2;1* injected oocytes and 2 min for *SvNIP2;2* injected oocytes. Oocytes were viewed under a dissecting microscope (Nikon SMZ800 light microscope, Japan) at 2× magnification. The changes in volume were captured with a Vicam color camera (Pacific Communications, Australia) at 2× magnification and recorded with IC Capture 2.0 software (The Imagine Source, US) as AVI format video files. Images were acquired every 2.5 s for 2 min measurements and every 2 s for 1 min measurements. The osmotic permeability (*P_f_*) was calculated for water injected and cRNA injected oocytes from the initial rate of change in relative volume (*dV*_rel_/*dt)_I_* determined from the cross sectional area images captured assuming the oocytes were spherical:

$$P_{\text{f}}=\frac{V_{\text{i}}\times {\left(dV_{\text{rel}}/dt\right)}_{\text{i}}}{A_{\text{i}}\times V_{\text{w}}\times \Delta C_{\text{0}}},$$

Where *V*_i_ and *A*_i_ are the initial volume and area of the oocyte, respectively, *V*_w_ is the partial molar volume of water and Δ*C*_o_ is the change in external osmolality. The osmolality of each solution was determined using a Fiske^®^ 210 Micro-Sample freezing point osmometer (Fiske, Norwood, MA, USA). pH inhibition of oocyte osmotic permeability was determined as above where oocytes where bathed in 1:5 diluted ND96 solution with the addition of 50 mM Na-Acetate, pH 5.6. Topological prediction models of SvPIP2;1 and SvNIP2;2 were generated in TMHMM^4^ (Krogh et al., 2001) and TMRPres-2D (Spyropoulos et al., 2004) to assess potential mechanisms of pH gating.

## Results

### Identification of Putative *Setaria viridis* Aquaporins

**FIGURE 1:**
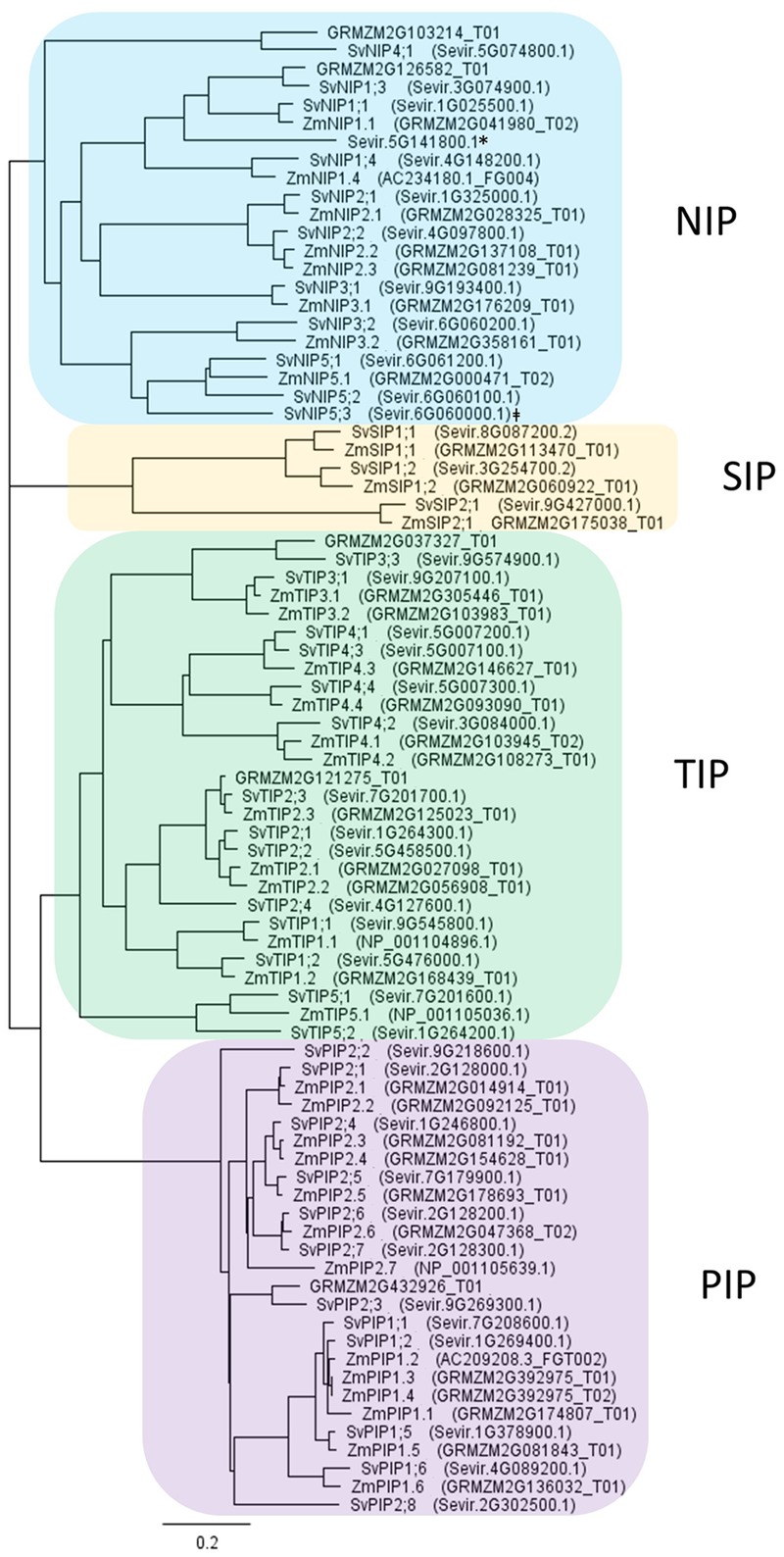
**Phylogenetic tree based on protein sequences of aquaporins from *Setaria viridis* and *Zea mays*.** *S. viridis* aquaporins were identified in the genome via HMMER search using aquaporins sequences from *Arabidopsis*, barley, maize, and rice. Maize aquaporins were included in the phylogenetic tree for ease of interpretation. The addition of aquaporin sequences from other grasses did not change the groupings. Tree was generated by neighbor-joining method using the Geneious Tree Builder program, Geneious 9.0.2. The scale bar indicates the evolutionary distance, expressed as changes per amino acid residue. Aquaporins can be grouped into four subfamilies: PIPs (plasma membrane intrinsic proteins), TIPs (tonoplast intrinsic proteins), NIPs (nodulin-like intrinsic proteins), and SIPs (small basic intrinsic proteins). ^∗^Sevir.5G141800.1 protein sequence is truncated, 112 amino acids in length. ^‡^SvNIP5;3 (Sevir.6G06000.1) may have a related pseudogene Sevir.6G061300.1.

Previously published *S. viridis* elongating internode transcriptome data (Martin et al., 2016), and protein sequences of aquaporins identified in *Arabidopsis, S. italica*, barley, maize and rice were used to identify genes predicted to encode aquaporins that were highly expressed in stages of cell expansion and sugar accumulation. The nomenclature assigned to the putative aquaporins followed their relative homology to previously named maize aquaporins determined by phylogenetic analysis of protein sequences (Chaumont et al., 2001; **Figure 1**). *S. viridis* proteins separated as expected into the major aquaporin subfamilies referred to as PIPs, TIPs, NIPs, and SIPs. Within *S. viridis* 41 full length aquaporins were identified: 12 PIPs, 14 TIPs, 12 NIPs, and three SIPs. One predicted aquaporin identified in the genome, transcript Sevir.6G061300.1, has very high similarity to SvNIP5;3 (Sevir.6G06000.1) but may be a pseudogene as it has two large deletions in the transcript relative to SvNIP5;3. Sevir.6G061300.1 only encodes for two out of the typical six transmembrane domains characteristic of aquaporins, and no transcripts have been detected in any of the *S. viridis* RNA-seq libraries available through the Joint Genome Institute (JGI) Plant Gene Atlas Project (Grigoriev et al., 2011). Another truncated NIP-like transcript, Sevir.5G141800.1, was identified. It is predicted to encode a protein 112 amino acids in length with only two transmembrane domains. As it is unlikely to generate an individually functioning aquaporin it has not been named. However, unlike Sevir.6G061300.1, Sevir.5G141800.1 was included in the phylogenetic tree as it was shown to be highly expressed in several tissue types in *S. viridis* RNA-seq libraries available through the JGI Plant Gene Atlas Project (Grigoriev et al., 2011) and may be of interest to future studies of *Setaria* aquaporin-like genes.

### Analysis of *Setaria viridis* Aquaporin Transcripts in Stem Regions

**FIGURE 2:**
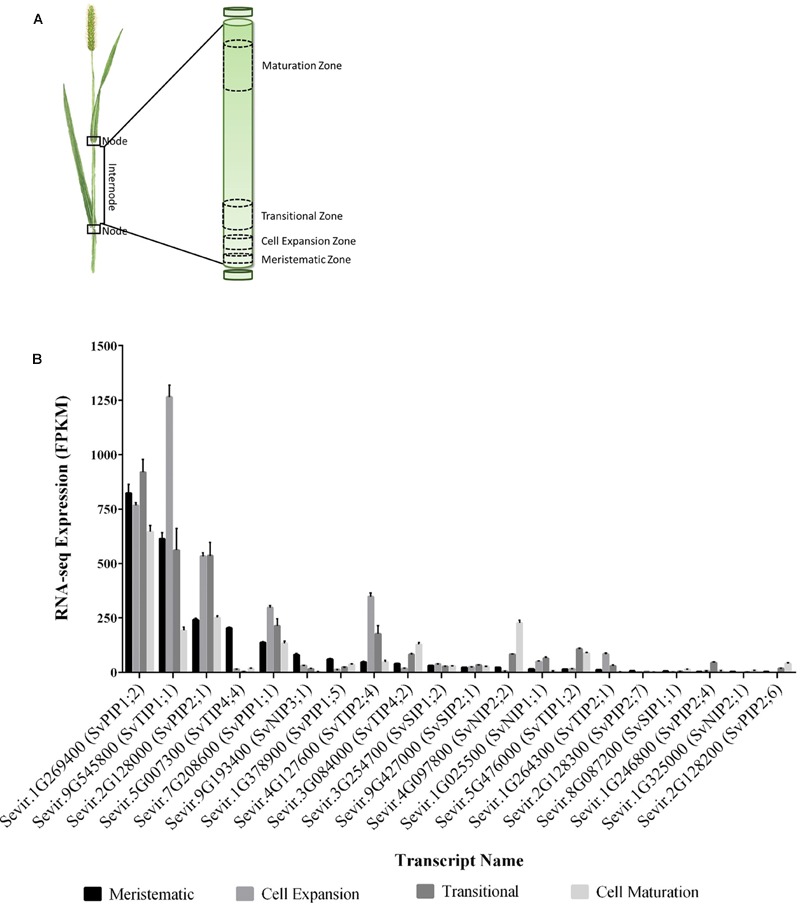
**Expression of putative aquaporins across the developmental zones of an elongating *S. viridis* internode. (A)** Schematic of the developmental regions in an elongating internode of *S. viridis* as reported by Martin et al. (2016): meristematic zone, residing at the base of the internode, where cell division occurs; the cell expansion zone where cells undergo turgor driven expansion; transitional zone where cells begin to differentiate and synthesize secondary cell walls; and the maturation zone whereby expansion, differentiation and secondary cell wall synthesis cease and sugar is accumulated. **(B)** The expression profiles of putative *S. viridis* aquaporins, as identified by phylogeny to *Z. mays* aquaporins, were mined in the *S. viridis* elongating internode transcriptome (Martin et al., 2016). RNA-seq data is presented as mean FPKM ± SEM for four biological replicates from each developmental zone.

We compared the relative transcript levels of putative *S. viridis* aquaporin encoding genes in the different developmental regions of an elongating internode (**Figure 2**). We observed that *SvPIP1;2* transcripts were abundant in all regions; and *SvTIP1;1* transcripts were also abundant, particularly in cell expansion regions. *SvPIP2;1, SvPIP1;1, SvTIP2;2*, and *SvTIP2;1* transcripts were detected in all regions with the highest transcript levels in cell expansion and transitional regions. Transcripts for *SvTIP4;4, SvNIP3;1*, and *SvPIP1;5* were highest in the meristem relative to other regions; whereas *SvTIP4;2, SvNIP2;2*, and *SvTIP1;2* transcripts were at their highest in transitional or mature regions. Low transcript levels were observed for *SvSIP1;2, SvNIP1;1*, and *SvPIP2;4* in all regions, with maximum transcripts for *SvNIP1;1* and *SvPIP2;4* detected in the transitional region, and very low transcript levels were detected for *SvPIP2;6, SvSIP1;1*, and *SvNIP2;1*.

Overall the highest aquaporin transcript levels detected across the internode developmental zones were those of *SvPIP1;2* (**Figure 2**). Previous research has indicated that the related ZmPIP1;2 interacts with PIP2 subgroup proteins targeting PIP2s to plasma membrane, and a number of PIP1 aquaporins are not associated with osmotic water permeability when expressed alone in oocytes (Fetter et al., 2004; Luu and Maurel, 2005; Zelazny et al., 2007). Our interest lay in identifying water permeable aquaporins that might be preferentially involved in delivering water to the growing stem cells and in sucrose accumulation in mature stem regions. As candidates *SvPIP2;1* and *SvNIP2;2* met these criteria we focussed on these two genes. *SvPIP2;1* had the high transcript levels in the region of cell expansion and transcript levels of *SvNIP2;2* were highest in mature stem regions (**Figure 2B**). The protein sequences of *SvPIP2;1* and *SvNIP2;2* were analyzed by the HMMER tool HMMscan which identified these candidates as belonging to the aquaporin (Major Intrinsic Protein) protein family.

**FIGURE 3:**
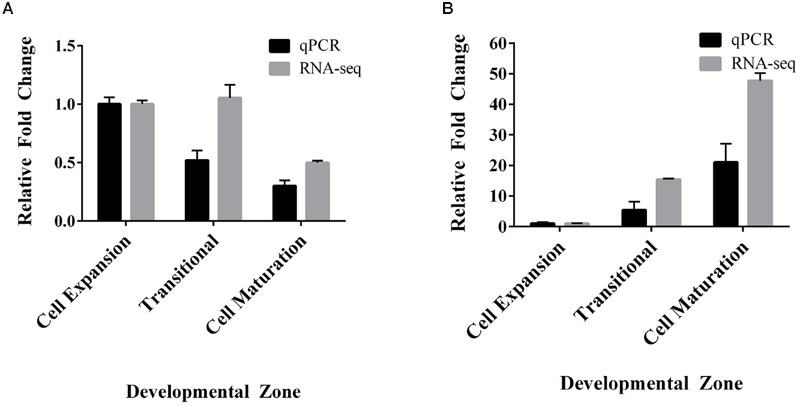
**Comparison of relative fold changes between RNA-seq and RT-qPCR of *SvPIP2;1* and *SvNIP2;2* in an elongating internode of S. viridis. (A)** *SvPIP2;1*. **(B)** *SvNIP2;2*. Data is mean relative fold change in expression ± SEM. Data for RNA-seq and RT-qPCR was normalized relative to the cell expansion zone expression level.

To confirm our RNA-seq expression profile observations, we measured the transcript levels of *SvPIP2;1* and *SvNIP2;2* in the *S. viridis* internode regions by RT-qPCR. Stem samples were harvested from *S. viridis* plants grown under glasshouse conditions with the light period artificially supplemented by use of metal halide lamps to replicate as closely as possible the conditions used by Martin et al. (2016) for the RNA-seq analysis. We assessed the relative fold change of gene expression normalized to the cell expansion zone and similar trends were observed for the RT-qPCR expression data compared to the RNA-seq transcriptome data (**Figure 3**). *SvPIP2;1* transcript levels were high in the cell expansion region and decreased toward the maturation region and *SvNIP2;2* transcript levels were highest in mature stem tissues.

**FIGURE 4:**
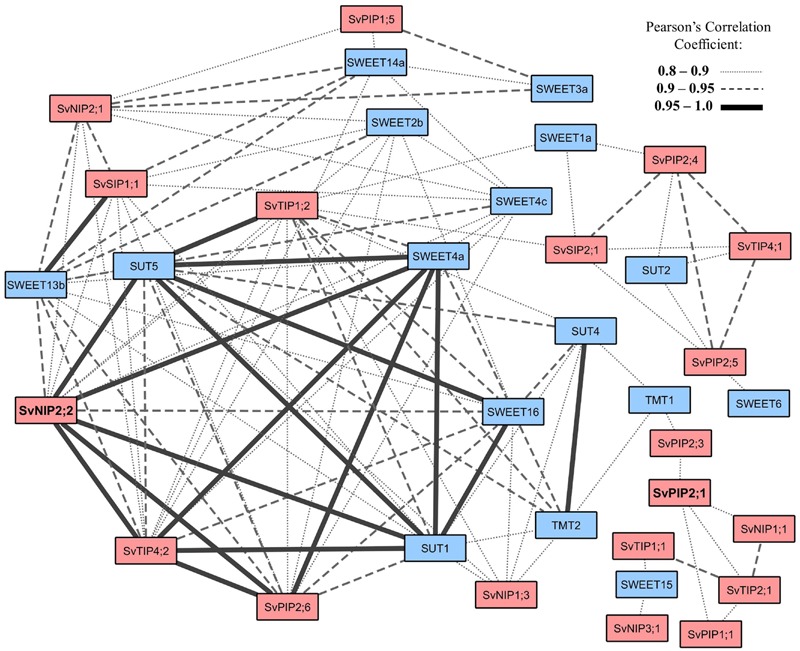
**Co-expression network of putative *S. viridis* aquaporin and sugar transporter genes identified in an elongating internode.** The co-expressed gene network was generated from the stem specific aquaporins (**Figure 2**) and sugar transporters identified in the *S. viridis* elongating internode transcriptome reported by Martin et al. (2016). Raw FPKM values were Log_2_ transformed and Pearson’s correlation coefficients (0.8–1.0) were calculated in the MetScape app in Cytoscape v3.4.0. Sugar transporters in the *S. viridis* elongating internode were identified by homology to rice sugar transporter genes (Supplementary Figures S2–S4). Sugar transport related genes are color filled with blue and aquaporin genes with orange. *SvNIP2;2* and *SvPIP2;1* are in bold font.

We are interested in the coordination of water and sugar transport related processes in developing grass stems. As a tool to investigate this, we further analyzed the stem transcriptome data to test whether any aquaporin and sugar transport related genes were co-expressed. Putative *S. viridis* sugar transporters were identified from the internode transcriptome (Martin et al., 2016) by homology to the rice sugar transporter families: SUTs, SWEETs, and TMTs (Supplementary Figures S2–S4). A co-expression gene network of the aquaporins and sugar transporters expressed in the *S. viridis* stem was generated in Cytoscape v3.4.0 using Pearson’s correlation coefficients calculated by MetScape (Karnovsky et al., 2012) (**Figure 4**). This analysis revealed that for a number of aquaporins and sugar transport related genes there was a high correlation in expression: *SvPIP2;1* expression correlated with the expression of *SvPIP2;3, SvTIP2;1*, and *SvNIP1;1* (0.8–0.9); and the correlation coefficients for co-expression of *SvPIP2;1* with *SvPIP2;5, SvTIP4;1, SvTIP1;2*, and *SWEET1a* were in the range of 0.8–0.9. Most notable was the high correlation (0.95–1.0) of expression of *SvNIP2;2* with sugar transport related genes *SvSUT5, SvSUT1, SvSWEET4a* and with *SvTIP4;2* and *SvPIP2;6*. The correlation between expression of *SvNIP2;2* and *SvSWEET13b* and *SvSWEET16* was also high (0.9–0.95). The *cis*-acting regulatory elements of the promoter regions of the aquaporin candidates *SvNIP2;2* and *SvPIP2;1*, and the putative sugar transporter genes *SvSUT1, SvSUT5*, and *SvSWEET4a* were analyzed (Supplementary Figure S5). There was no obvious relationship between the correlation of expression of *SvNIP2;2* and *SvSUT1, SvSUT5* and *SvSWEET4a* and their *cis-*acting regulatory elements.

### Characterisation of *Setaria viridis* PIP2;1 and NIP2;2 in *Xenopus laevis* Oocytes

**FIGURE 5:**
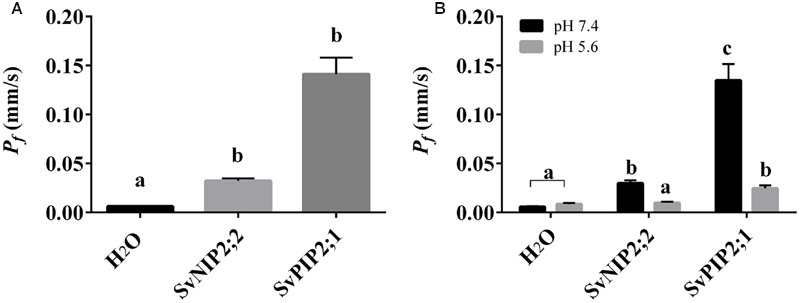
**Osmotic permeability (*P*_f_) of *Xenopus laevis* oocytes injected with *SvNIP2;2* and *SvPIP2;1* cRNA. (A)** Osmotic permeability (*P*_f_) of water (H_2_O) injected and *SvNIP2;2* and *SvPIP2;1* cRNA (46 ng) injected oocytes. Oocytes were transferred into a hypo-osmotic solution, pH 7.4, and *P*_f_ was calculated by video monitoring of the rate of oocyte swelling. **(B)** Effect of lowering oocyte cytosolic pH on osmotic permeability (*P*_f_) of H_2_O and *SvNIP2;2* and *SvPIP2;1* cRNA injected oocytes by bathing in hypo-osmotic solution supplemented with 50 mM Na-acetate, pH 5.6, *n* = 12–14; a = non-significant; b = *p* < 0.05; c = *p* < 0.005.

To explore whether the proteins encoded by *SvPIP2;1* and *SvNIP2;2* function as water channels they were expressed in the heterologous *X. laevis* oocytes system. Water with or without 46 ng of *SvPIP2;1* and *SvNIP2;2* cRNA was injected into oocytes and the swelling of these oocytes in response to bathing in a hypo-osmotic solution (pH 7.4) was measured (**Figure 5A**). The osmotic permeability (*P_f_*) of cRNA injected oocytes was calculated and compared to the osmotic permeability of water injected oocytes. Water injected oocytes had a *P_f_* of 0.60 ± 0.08 × 10^-2^ mm s^-1^. Relative to water injected control oocytes *SvPIP2;1* and *SvNIP2;2* cRNA injected oocytes had significantly higher *P_f_* of 14.13 ± 1.66 × 10^-2^ mm s^-1^ and 3.22 ± 0.28 × 10^-2^ mm s^-1^, respectively (*p* < 0.05).

The effect of lowering oocyte cytosolic pH was determined by bathing oocytes in an external hypo-osmotic solution at pH 5.6 with the addition of Na-Acetate (**Figure 5B**). Reduced osmotic permeability of the cRNA injected oocyte membrane was observed in response to the low pH treatment. A reduction in *P*_f_ was observed for *SvPIP2;1* and *SvNIP2;2* cRNA injected oocytes bathed in an external hypo-osmotic solution at pH 5.6 relative to the pH 7.4 solution indicating that SvPIP2;1 and SvNIP2;2 have pH gating mechanisms (**Figure 5B**). Water injected oocytes in the pH 5.6 Na-Acetate solution had *P*_f_ of 0.84 ± 0.13 × 10^-2^ mm s^-1^. *SvNIP2;2* and *SvPIP2;1* cRNA injected oocytes in the pH 5.6 solution had significantly lower *P_f_* of 2.46 ± 0.32 × 10^-2^ mm s^-1^ and 0.97 ± 0.13 × 10^-2^ mm s^-1^, respectively, compared to those in pH 7.4 solution (*p* < 0.05). SvPIP2;1 and SvNIP2;2 associated osmotic permeability and pH gating observations indicate that these proteins can function as water channels. The mechanism of pH gating for other plant aquaporins is the protonation of a Histidine residue in the Loop D structure; topological modeling of SvPIP2;1 and SvNIP2;2 predicted that the Loop D of SvPIP2;1 contains a Histidine residue while SvNIP2;2 Loop D does not contain a His residue (Supplementary Figure S6).

## Discussion

### Roles of Aquaporins in Grass Stem Development

On the basis of amino acid sequence comparison with known aquaporins in *Arabidopsis*, rice and maize, the genomes of sugarcane, sorghum and *S. italica* include 42, 41, and 42 predicted aquaporin encoding genes, respectively (da Silva et al., 2013; Reddy et al., 2015; Azad et al., 2016). In *S. viridis* 41 aquaporin encoding genes were identified that group into four clades corresponding to NIPs, TIPs, SIPs, and PIPs (**Figure 1**). We note that Azad et al. (2016) named the *Setaria* aquaporins in an order consecutive with where they are found in the genome. For ease of comparing related aquaporins in C_4_ grasses of interest, we named the *Setaria* aquaporins based on their homology to previously named maize aquaporins (**Figure 1**) (Chaumont et al., 2001), of course high homology and the same name does not infer the same function. In the *S. viridis* elongating internode transcriptome, we detected transcripts for 19 putative aquaporin encoding genes, including 5 NIPs, 6 TIPs, 2 SIPs, and 6 PIPs (**Figures 2** and **3**; Martin et al., 2016). In mature *S. viridis* internode tissues, the transcript levels of TIPs and NIPs was generally low with the exception of *SvNIP2;2, SvTIP4;2*, and *SvTIP1;2.* In a sorghum stem transcriptome report investigating SWEET gene involvement in sucrose accumulation, we note that transcripts for all 41 sorghum aquaporins were detected in pith and rind tissues in 60-day-old plants (Reddy et al., 2015; Mizuno et al., 2016). Of those 41 aquaporins the expression of 16, primarily NIPs and TIPs, was relatively low. However, *PIP1;2, PIP2;1*, and *NIP2;2* homologs were all highly expressed in pith and rind of sorghum plants after heading, which is consistent with our findings for the *S. viridis* homologs of these genes (**Figure 2**; Mizuno et al., 2016). Comparisons with other gene expression studies for C_4_ grass stem tissues were not possible as in most studies the internode tissue has not been separated into different developmental zones or the study has not reported aquaporin expression (Carson and Botha, 2000, 2002; Casu et al., 2007).

### Relationships between Sink Strength, Sink Size, Water Flow, and the Function of Aquaporins

The molecular and physiological mechanisms that determine stem cell number and cell size in turn determine the capacity of the stem as a sink (Ho, 1988; Herbers and Sonnewald, 1998). Examples have been reported in the literature where stem volume and sucrose concentration has been increased, in sugarcane and sorghum, by increasing cell size (Slewinski, 2012; Patrick et al., 2013). Larger cell size may improve sink strength by increasing membrane surface area available to sucrose transport (increasing import capacity), increasing single cell capacity to accumulate greater concentrations of sucrose in parenchyma cell vacuoles due to increased individual cell volume (increasing storage capacity), and increasing lignocellulosic biomass.

Cell expansion and growth are highly sensitive to water potential. This is because expansion requires a continuous influx of water into the cell to maintain turgor pressure (Hsiao and Acevedo, 1974; Cosgrove, 1986, 2005). The diffusion of water across a plant cell membrane is facilitated by aquaporins (Kaldenhoff and Fischer, 2006). Aquaporins function throughout all developmental stages, but several PIP aquaporins have been found to be particularly highly expressed in regions of cell expansion (Chaumont et al., 1998; Maurel et al., 2008; Besse et al., 2011). Here, we report that in the *S. viridis* internode, *SvPIP2;1* was highly expressed in regions undergoing cell expansion (**Figure 2**). Positive correlations have been reported for the relationship between PIP mRNA and protein expression profiles of PIP isoforms in the expanding regions of embryos, roots, hypocotyls, leaves, and reproductive organs indicating that gene expression is a key mechanisms to regulate PIP function (Maurel et al., 2002; Hachez et al., 2008; Liu et al., 2008). Therefore, high expression of *SvPIP2;1* in the expanding zone of *S. viridis* internodes indicates that this gene may be involved in the process of water influx in this tissue to maintain turgor pressure for growth.

The roles of a number of PIP proteins in hydraulic conductivity in plant roots and leaves have been reported but PIP function in stems is largely unexplored. The regulation of the hydraulic properties of expanding root tissues by PIP expression was analyzed by Péret et al. (2012) and they reported that auxin mediated reduction of *Arabidopsis thaliana* (At) PIP gene expression resulted in delayed lateral root emergence. Previously *AtPIP2;2* anti-sense mutants were reported to have lower (25–30%) hydraulic conductivity of root cortex cells than control plants (Javot et al., 2003). PIP2 family aquaporins, involved in cellular water transport in roots have also been linked to water movement in leaves, seeds, and reproductive organs (Schuurmans et al., 2003; Bots et al., 2005). The roles of PIP proteins in maintenance of hydraulic conductivity and cell expansion in stems are likely to be equally as important as the roles reported for PIPs in the expanding tissues of roots and leaves. One study in rice reported OsPIP1;1 and OsPIP2;1 as being highly expressed in the zone of cell expansion in rapidly growing internodes (Malz and Sauter, 1999). Expression analysis of sugarcane genes associated with sucrose content identified that some unnamed PIP isoforms were highly expressed in immature internodes, and in high sugar yield cultivars (Papini-Terzi et al., 2009). Proteins from the PIP2 subfamily in particular in maize, spinach and *Arabidopsis* have been shown to be highly permeable to water (Johansson et al., 1998; Chaumont et al., 2000; Kaldenhoff and Fischer, 2006). Here, we demonstrate, by expression of SvPIP2;1 in *Xenopus* oocytes and analysis of water permeability, that this protein functions as a water channel (**Figure 5A**).

### Aquaporin Function and Sugar Accumulation in Mature Grass Stems

The accumulation of sucrose to high concentrations in panicoid stems rapidly increases with the cessation of cell expansion, which is also associated with the deposition of secondary cell walls (Hoffmann-Thoma et al., 1996). In the mature regions of the stem internodes, imported sucrose is no longer required for growth, development, or as a necessary precursor to structural elements and it is stored in the vacuoles of ground parenchyma cells or the apoplasm (Rae et al., 2009). Phloem unloading and the delivery of sucrose to these storage cells may occur via an apoplasmic pathway as in sorghum or a symplasmic pathway as in sugarcane (Welbaum and Meinzer, 1990; Walsh et al., 2005). The degree of suberisation and/or lignification of cell walls surrounding the phloem may influence stem sucrose storage traits by restricting apoplasmic pathways of sucrose transport. In potato tubers and *Arabidopsis* ovules a switch between apoplasmic and symplasmic pathways of delivering sucrose to storage sites has been reported (Viola et al., 2001; Werner et al., 2011). Similarly, a switch from symplasmic to apoplasmic transport pathways has been proposed for sorghum as internodes approach maturity (Tarpley et al., 2007; Milne et al., 2015). Both apoplasmic and symplasmic mechanisms of phloem unloading require the maintenance of low sugar concentration in the cytoplasm of parenchymal storage cells. Control of hydrostatic pressure is facilitated by the sequestration of sucrose into the vacuole by tonoplast localized SUTs or into the apoplasm by plasma membrane localized SUTs (Slewinski, 2011). Members of the SUT and TMT families have been shown to function on the tonoplast to facilitate sucrose accumulation in the vacuole (Reinders et al., 2008; Wingenter et al., 2010; Bihmidine et al., 2016). In mature stem tissue plasma membrane localized SWEETs, SUTs, and possibly some NIPs may have a role in transporting sugar into the apoplasm (Milne et al., 2013; Chen, 2014).

The cell maturation zone is characterized by cells that have ceased expansion and differentiation and have realized their sugar accumulation capacity (Rohwer and Botha, 2001; McCormick et al., 2009). In mature sink tissues, the movement of water and dissolved photoassimilates from the phloem to storage parenchyma cells may be driven by differences in solute concentration and hydrostatic pressure (Turgeon, 2010; De Schepper et al., 2013). However, the movement of water and sucrose by diffusion or bulk-flow requires the continued maintenance of low cytosolic sucrose concentrations by accumulation of sucrose into the vacuole or eﬄux into the apoplasm for storage (Grof et al., 2013). Throughout internode development, the internal cell pressure of storage parenchyma cells in sugarcane remains relatively constant despite increasing solute concentrations toward maturation (Moore and Cosgrove, 1991). As mature cells tend to have heavily lignified cell walls that limit the ability of the protoplast to expand in response to water flux the equilibration of storage parenchyma cell turgor is likely to be achieved by the partitioning of sucrose into the vacuole and apoplasm, and eﬄux of water into the apoplasm (Moore and Cosgrove, 1991; Vogel, 2008; Keegstra, 2010; Moore and Botha, 2013). Phloem water eﬄuxed into the apoplasm may then be recycled back to the vascular bundles (Welbaum et al., 1992).

Members of the NIPs are candidates for water and neutral solute permeation, and some NIPs could have a role in water and solute eﬄux to the apoplasm in mature stem cells (Takano et al., 2006; Kamiya et al., 2009; Li et al., 2009; Hanaoka et al., 2014). The NIP subfamily is divided into the subgroups NIP I, NIP II, and NIP III based on the composition of the ar/R selectivity filter (Liu and Zhu, 2010). NIP III subgroup homologs have reported permeability to water, urea, boric acid, and silicic acid (Bienert et al., 2008; Ma et al., 2008; Ma and Yamaji, 2008; Li et al., 2009). In grasses NIP2;2 homologs, from the NIP III subgroup, have been shown to localize to the plasma membrane (Ma et al., 2006).

In the *S. viridis* internode, *SvNIP2;2* had relatively high transcript levels in mature stem tissue where sugar accumulates, and it can function as a water channel, although with a relatively low water permeability compared to SvPIP2;1 (**Figures 2** and **5A**). Our analysis of gene co-expression in stem tissues revealed high correlation between the expression of *SvNIP2;2* and five putative *S. viridis* sugar transporter genes (**Figure 4**). Co-expression can indicate that genes are controlled by the same transcriptional regulatory program, may be functionally related, or be members of the same pathway or protein complex (Eisen et al., 1998; Yonekura-Sakakibara and Saito, 2013). The strong correlation between expression of *SvNIP2;2* and key putative sugar transport related genes such as *SvSUT5, SvSUT1, SvSWEET4a, SvSWEET13b*, and *SvSWEET16* indicates that they may be involved in a related biological process such as stem sugar accumulation. It is likely that one or more of the SWEETs have roles in transporting sugars out of the stem parenchyma cells into the apoplasm. SvNIP2;2 may be permeable to neutral solutes as well as water and the role of this protein in the mature stem could be in eﬄuxing a solute to adjust osmotic pressure allowing for greater sugar storage capacity. The rice and soybean (*Glycine max* L.) NIP2;2 proteins are permeable to silicic acid and silicon, respectively (Ma et al., 2006; Zhao et al., 2010; Deshmukh et al., 2013). The deposition of silicic acid into the apoplasm, where it associates with the cell wall matrix as a polymer of hydrated amorphous silica (Epstein, 1994; Ma et al., 2004; Coskun et al., 2016), strengthens the culm to reduce lodging events, and increases plant resistance to pathogens and abiotic stress factors (Mitani, 2005).

SvNIP2;2 water permeability was gated by pH (**Figure 5B**). Gating of water channel activity has been reported for PIPs, including SvPIP2;1 (**Figure 5B**), and for the TIP2;1 isoform found in grapevine (Törnroth-Horsefield et al., 2006; Leitao et al., 2012; Frick et al., 2013). The mechanism of pH gating for these AQPs is the protonation of a Histidine residue located on the cytoplasmic Loop D where site-directed mutagenesis studies of the Loop D His residue results in a loss of pH dependent water permeability (Tournaire-Roux et al., 2002; Leitao et al., 2012; Frick et al., 2013). However, although SvNIP2;2 water permeability was pH dependent the predicted Loop D structure does not contain a His residue (Supplementary Figure S5), hence for SvNIP2;2 the mechanism for pH gating is not clear.

## Conclusion

Our observations of high transcript levels of *SvPIP2;1* in expanding *S. viridis* stem regions and high transcript levels of *SvNIP2;2* in mature stems inspired us to test the function of the proteins encoded by these genes. We found that SvPIP2;1 and SvNIP2;2 can function as pH gated water channels. We hypothesize that in stem tissues SvPIP2;1 is involved in cell growth and that SvNIP2;2 may facilitate water movement and potentially the flow of other solutes into the apoplasm to sustain solute transportation by bulk-flow, and possibly ‘recycle’ water used for solute delivery back to the xylem. It is expected that SvPIP2;1 could have additional roles, as other PIP water channels have been shown to also be permeable to CO_2_, hydrogen peroxide, urea, sodium and arsenic (Siefritz et al., 2001; Uehlein et al., 2003; Mosa et al., 2012; Bienert and Chaumont, 2014; Byrt et al., 2016b). SvNIP2;2 could have roles such as transporting neutral solutes to the apoplasm, as previous studies report silicic acid, urea, and boric acid permeability for other NIPS (Bienert et al., 2008; Ma et al., 2008; Ma and Yamaji, 2008; Li et al., 2009; Deshmukh et al., 2013). Transporting solutes other than sucrose into the apoplasm in mature stem tissues may be an important part of the processes that supports high sucrose accumulation capacity in grass stem parenchyma cells. The next steps in establishing the respective functions of SvPIP2;1 and SvNIP2;2 in stem growth and sugar accumulation in *S. viridis* will require testing of the permeability of these proteins to a range of other solutes and modification of their function *in planta*.

## Author Contributions

CG conceived and designed the work. SM, HO, LC, and JP acquired the data. SM, ST, CB, and CG analyzed and interpreted the data. SM and CB drafted and revised the work. All authors commented on the manuscript. SM, ST, RF, CB, and CG revised the work critically for intellectual content.

## Conflict of Interest Statement

The authors declare that the research was conducted in the absence of any commercial or financial relationships that could be construed as a potential conflict of interest.

## Footnotes

1 http://www.ebi.ac.uk/Tools/hmmer/

2 http://phytozome.jgi.doe.gov/

3 http://bioinformatics.psb.ugent.be/webtools/plantcare/html/

4 http://www.cbs.dtu.dk/services/TMHMM/
