# Supplementary material for: Roles of Aquaporins in Setaria viridis Stem Development and Sugar Storage
Source: Front Plant Sci. 2016 Dec 1;7:1815. doi: 10.3389/fpls.2016.01815 (PMC5147461; doi:10.3389/fpls.2016.01815)
Supplement: Supplementary file 1 [file Data_Sheet_1.DOCX]

| **Table S1: Summary of *Setaria italica* and *Setaria viridis* aquaporins.** | | | | | |  |  |
| --- | --- | --- | --- | --- | --- | --- | --- |
|  |  |  |  |  |  |  |  |
| *Setaria italica* | |  |  | *Setaria viridis* |  |  |  |
| **Name** | **Gene ID** | **Accession number** | **AA length** | **Name** | **Accession number** | **AA length** | **% protein identity *S. viridis* to *S. italica*** |
| SiPIP1;1 | Si010758m | Seita.7G196700 | 288 | SvPIP1;1 | Sevir.7G208600 | 288 | 100 |
| SiPIP1;2 | Si017731m | Seita.1G264900 | 337 | SvPIP1;2 | Sevir.1G269400 | 289 | 82.1 |
| SiPIP1;5 | Si017991m | Seita.1G372300 | 288 | SvPIP1;5 | Sevir.1G378900 | 288 | 100 |
| SiPIP1;6 | Si007002m | Seita.4G089800 | 299 | SvPIP1;6 | Sevir.4G089200 | 299 | 100 |
| SiPIP2;1 | Si030703m | Seita.2G123000 | 289 | SvPIP2;1 | Sevir.2G128000 | 289 | 100 |
| SiPIP2;2 | Si039883m | Seita.9G219400 | 294 | SvPIP2;2 | Sevir.9G218600 | 294 | 100 |
| SiPIP2;3 | Si036923m | Seita.9G268100 | 284 | SvPIP2;3 | Sevir.9G269300 | 284 | 100 |
| SiPIP2;4 | Si017990m | Seita.1G241900 | 288 | SvPIP2;4 | Sevir.1G246800 | 290 | 99.3 |
| SiPIP2;5 | Si010750m | Seita.7G170200 | 290 | SvPIP2;5 | Sevir.7G179900 | 290 | 100 |
| SiPIP2;6 | Si030713m | Seita.2G123200 | 286 | SvPIP2;6 | Sevir.2G128200 | 286 | 100 |
| SiPIP2;7 | Si030712m | Seita.2G123300 | 286 | SvPIP2;7 | Sevir.2G128300 | 286 | 100 |
| SiPIP2;8 | Si030718m | Seita.2G291500 | 285 | SvPIP2;8 | Sevir.2G302500 | 285 | 100 |
| SiTIP1;1 | Si037174m | Seita.9G541300 | 249 | SvTIP1;1 | Sevir.9G545800 | 249 | 100 |
| SiTIP1;2 | Si004276m | Seita.5G469800 | 268 | SvTIP1;2 | Sevir.5G476000 | 252 | 93.7 |
| SiTIP2;1 | Si018184m | Seita.1G259900 | 249 | SvTIP2;1 | Sevir.1G264300 | 249 | 100 |
| SiTIP2;2 | Si004690m | Seita.5G452400 | 305 | SvTIP2;2 | Sevir.5G458500 | 244 | 77 |
| SiTIP2;3 | Si010925m | Seita.7G189600 | 248 | SvTIP2;3 | Sevir.7G201700 | 248 | 100 |
| SiTIP2;4 | Si007145m | Seita.4G160700 | 252 | SvTIP2;4 | Sevir.4G127600 | 248 | 98 |
| SiTIP3;1 | Si037080m | Seita.9G208400 | 262 | SvTIP3;1 | Sevir.9G207100 | 262 | 100 |
| SiTIP3;2 | Si012227m | Seita.7G175600 | 318 |  |  |  |  |
| SiTIP3;3 | Si037121m | Seita.9G571600 | 256 | SvTIP3;3 | Sevir.9G574900 | 256 | 92.2 |
| SiTIP4;1 | Si002696m | Seita.5G007400 | 246 | SvTIP4;1 | Sevir.5G007200 | 246 | 99.6 |
| SiTIP4;2 | Si022367m | Seita.3G082100 | 377 | SvTIP4;2 | Sevir.3G084000 | 259 | 99.2 |
| SiTIP4;3 | Si003768m | Seita.5G007300 | 246 | SvTIP4;3 | Sevir.5G007100 | 246 | 100 |
| SiTIP4;4 | Si002674m | Seita.5G007500 | 250 | SvTIP4;4 | Sevir.5G007300 | 295 | 82.4 |
| SiTIP5;1 | Si011789m | Seita.7G189500 | 268 | SvTIP5;1 | Sevir.7G201600 | 268 | 100 |
| SiTIP5;2 | Si019510m | Seita.1G259800 | 259 | SvTIP5;2 | Sevir.1G264200 | 259 | 100 |
| SiNIP1;1 | Si018035m | Seita.1G025100 | 278 | SvNIP1;1 | Sevir.1G025500 | 279 | 100 |
| SiNIP1;3 | Si022896m | Seita.3G073300 | 281 | SvNIP1;3 | Sevir.3G074900 | 282 | 100 |
| SiNIP1;4 | Si008400m | Seita.4G180100 | 286 | SvNIP1;4 | Sevir.4G148200 | 287 | 99.7 |
| SiNIP2;1 | Si017716m | Seita.1G318800 | 341 | SvNIP2;1 | Sevir.1G325000 | 297 | 100 |
| SiNIP2;2 | Si007007m | Seita.4G098700 | 297 | SvNIP2;2 | Sevir.4G097800 | 298 | 100 |
| SiNIP3;1 | Si036817m | Seita.9G193500 | 299 | SvNIP3;1 | Sevir.9G193400 | 300 | 100 |
| SiNIP3;2 | Si015338m | Seita.6G062400 | 291 | SvNIP3;2 | Sevir.6G060200 | 291 | 98.6 |
| SiNIP4;1 | Si002921m | Seita.5G076000 | 210 | SvNIP4;1 | Sevir.5G074800 | 299 | 100 |
| SiNIP5;1 | Si015217m | Seita.6G063300 | 285 | SvNIP5;1 | Sevir.6G061200 | 285 | 95.4 |
| SiNIP5;2 | Si015485m | Seita.6G062300 | 286 | SvNIP5;2 | Sevir.6G060100 | 287 | 99.7 |
| SiNIP5;3 | Si014171m | Seita.6G062200 | 296 | SvNIP5;3 | Sevir.6G060000 | 296 | 100 |
| SiNIP5;4 | Si015350m | Seita.6G063400.1 | 211 | SvNIP5;4 | Sevir.6G061300.1 | 212 | 62.1 |
| SiSIP1;1 | Si027934m | Seita.8G085300 | 200 | SvSIP1;1 | Sevir.8G087200 | 252 | 99.5 |
| SiSIP1;2 | Si023083m | Seita.3G248900 | 243 | SvSIP1;2 | Sevir.3G254700 | 244 | 100 |
| SiSIP2;1 | Si037152m | Seita.9G422800 | 252 | SvSIP2;1 | Sevir.9G427000 | 252 | 77.8 |

| **Table S2: Summary of *Zea mays* aquaporins.** | | |  |
| --- | --- | --- | --- |
|  |  |  |  |
| *Zea mays* |  |  |  |
| **Name** | **Accession number** | **Phytozome number** | **amino acid length** |
| ZmPIP1;1 | X82633 | GRMZM2G174807_T01 |  |
| ZmPIP1;2 | AF131201 | AC209208.3_FGT002 | 289 |
| ZmPIP1;3 | AF326487 | GRMZM2G392975_T01 | 292 |
| ZmPIP1;4 | AF326488 | GRMZM2G392975_T02 | 292 |
| ZmPIP1;5 | AF326489 | GRMZM2G081843_T01 | 288 |
| ZmPIP1;6 | AF326490 | GRMZM2G136032_T01 | 296 |
| ZmPIP2;1 | AF326491 | GRMZM2G014914_T01 | 290 |
| ZmPIP2;2 | AF326492 | GRMZM2G092125_T01 | 291 |
| ZmPIP2;3 | AF326493 | GRMZM2G081192_T01 | 289 |
| ZmPIP2;4 | AF326494 | GRMZM2G154628_T01 | 288 |
| ZmPIP2;5 | AF130975 | GRMZM2G178693_T01 | 285 |
| ZmPIP2;6 | AF326495 | GRMZM2G047368_T02 | 288 |
| ZmPIP2;7 | AF326496 |  | 287 |
| hypothetical |  | GRMZM2G432926_T01 | 283 |
|  |  |  |  |
| ZmTIP1;1 | AF037061 |  | 250 |
| ZmTIP1;2 | AF326500 | GRMZM2G168439_T01 | 254 |
| ZmTIP2;1 | AF326501 | GRMZM2G027098_T01 | 249 |
| ZmTIP2;2 | AF326502 | GRMZM2G056908_T01 | 250 |
| ZmTIP2;3 | AF326503 | GRMZM2G125023_T01 | 248 |
| ZmTIP3;1 | AF326504 | GRMZM2G305446_T01 | 262 |
| ZmTIP3;2 | AF342809 | GRMZM2G103983_T01 | 266 |
| ZmTIP4;1 | AF326505 | GRMZM2G103945_T02 | 255 |
| ZmTIP4;2 | AF326506 | GRMZM2G108273_T01 | 311 |
| ZmTIP4;3 | AF326507 | GRMZM2G146627_T01 | 249 |
| ZmTIP4;4 | AF326508 | GRMZM2G093090_T01 | 252 |
| ZmTIP5;1 | AF326509 |  | 260 |
| hypothetical | XP_008669054.1 | GRMZM2G121275_T01 | 248 |
| hypothetical | NP_001146930.1 | GRMZM2G037327_T01 | 267 |
|  |  |  |  |
| ZmNIP1;1 | AF326483 | GRMZM2G041980_T02 | 282 |
| ZmNIP1;4 |  | AC234180.1_FG004 | 284 |
| ZmNIP2;1 | AF326484 | GRMZM2G028325_T01 | 295 |
| ZmNIP2;2 | AF326485 | GRMZM2G137108_T01 | 294 |
| ZmNIP2;3 | AF342810 | GRMZM2G081239_T01 | 301 |
| ZmNIP3;1 | AF326486 | GRMZM2G176209_T01 | 302 |
| ZmNIP3;2 | XP_008664035.1 | GRMZM2G358161_T01 | 274 |
| ZmNIP5;1 | AFW61239.1 | GRMZM2G000471_T02 | 296 |
| hypothetical | ACL53915.1 | GRMZM2G103214_T01 | 299 |
| hypothetical | AFW77428.1 | GRMZM2G126582_T01 | 284 |
|  |  |  |  |
| ZmSIP1;1 | AF326497 | GRMZM2G113470_T01 | 245 |
| ZmSIP1;2 | AF326498 | GRMZM2G060922_T01 | 243 |
| ZmSIP2;1 | AF326499 | GRMZM2G175038_T01 | 249 |

**Figure S1: Expression of housekeeper gene PP2A (Seita.7G316700.1) across the developmental zones in the RT-qPCR and transcriptome data. (A)** RT-qPCR Ct values SvPP2A expression in the developmental zones of an elongating *S. viridis* internode. Data is average of three technical replicates over 4 biological replicates $\pm$ SEM. **(B)** Raw FPKM values of SvPP2A expression across the developmental zones in the *S. viridis* elongating internode transcriptome reported by Martin et al (2016). a; p >0.05, n.s

**
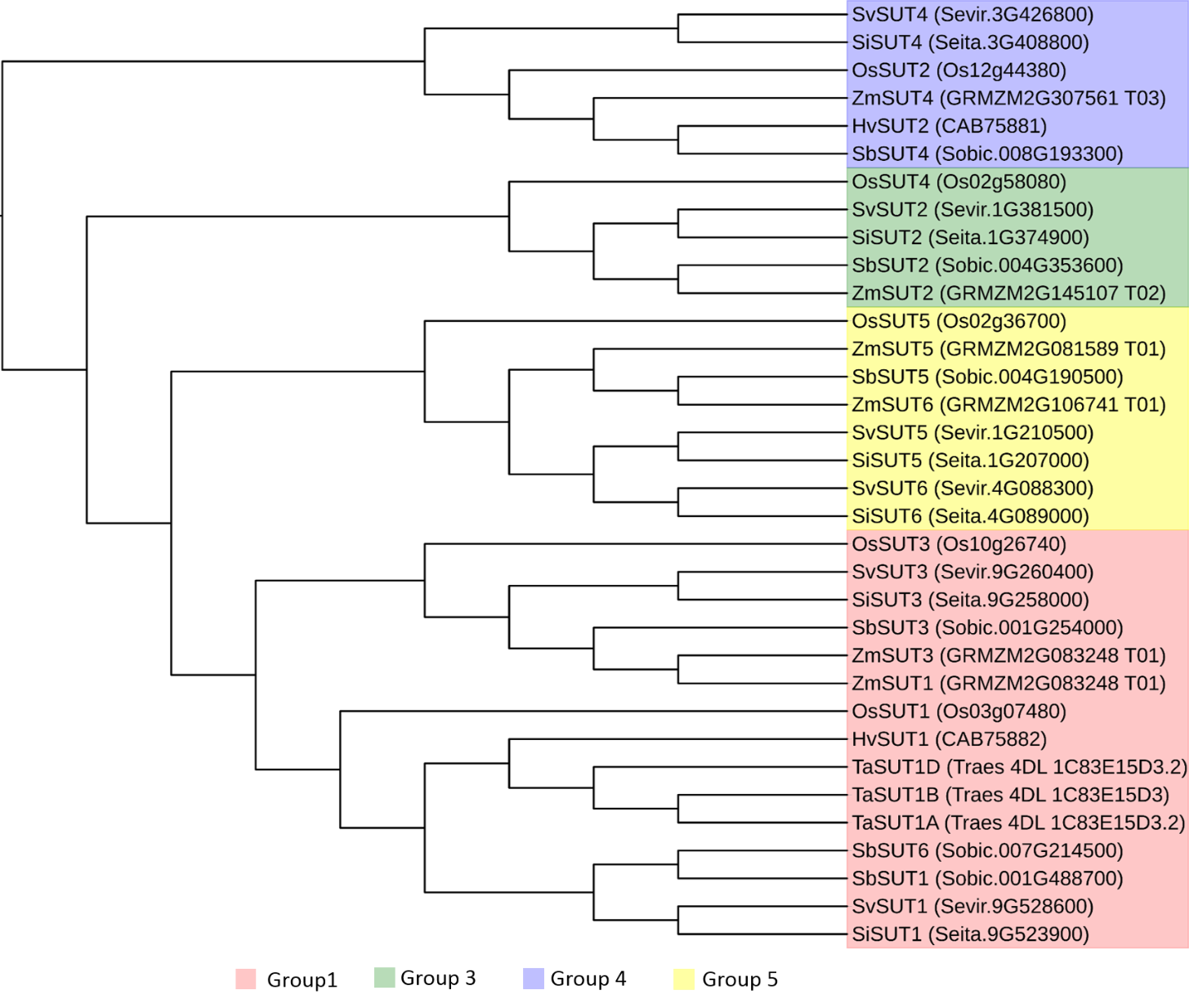
**

**Figure S2: Phylogeny of putative *S. viridis* and major monocotyledonous crop SUT proteins.** Monocotyledonous species included are; *Hordeum vulgare* (Hv), *Oryza sativa* (Os), *Sorghum bicolor* (Sb), *Setaria italica* (Si), *Setaria viridis* (Sv), *Triticum aesvitum* (Ta) and *Zea mays* (Zm). All accession numbers are from Phytozome v11 (<https://phytozome.jgi.doe.gov/pz/portal.html>) except for *Hordeum vulgare* which are from NCBI (http://www.ncbi.nlm.nih.gov/). Protein sequences of SUTs were aligned by MUSCLE and iTOL v.3 (http://itol.embl.de/) was used to generate the phylogenetic tree. Adapted from (Milne et al., 2013).

**
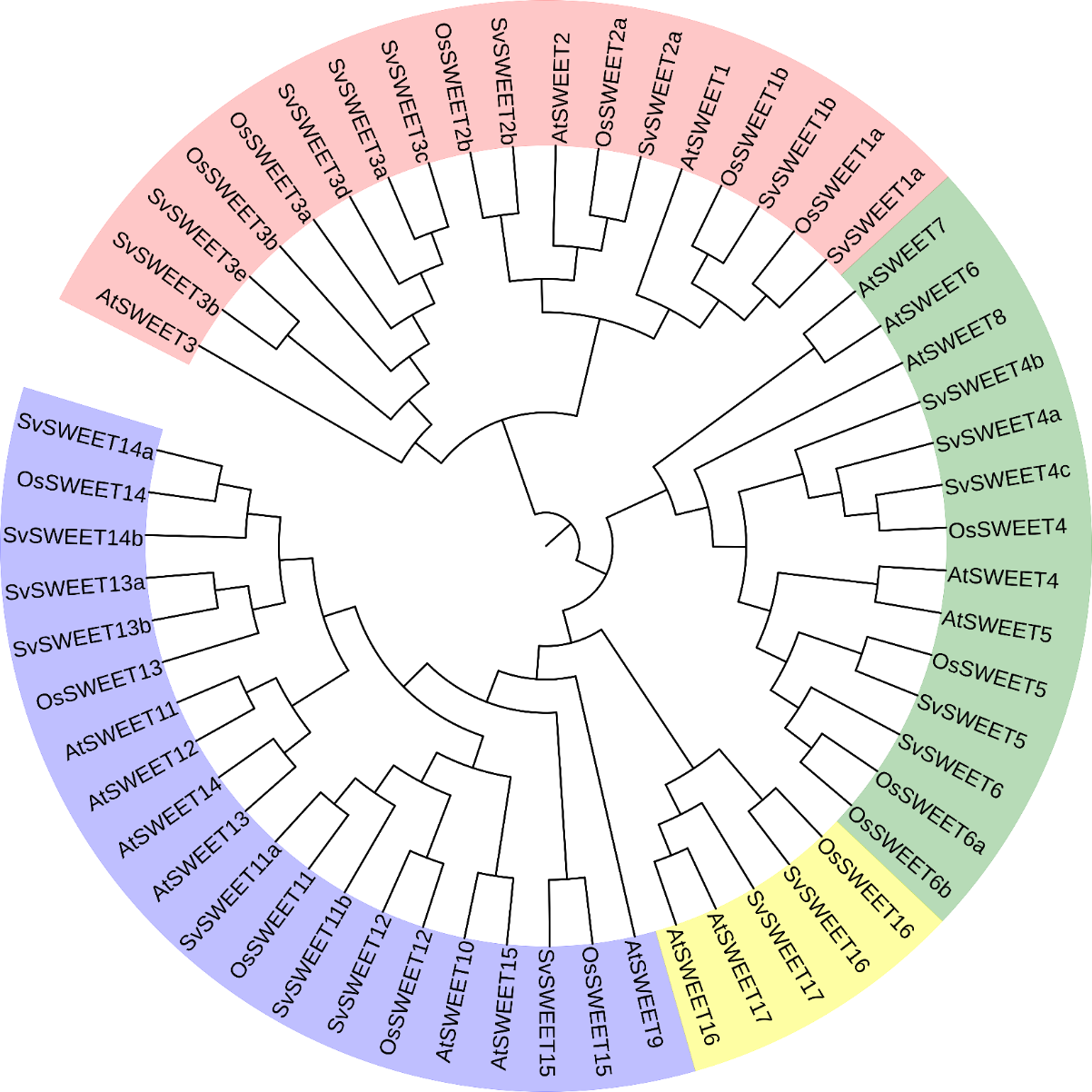
**

**Figure S3: Phylogeny and naming of putative *S. viridis* SWEET proteins.** Protein sequences of Arabidopsis and rice SWEET genes identified in (Chen et al., 2010) were BLAST in Phytozome v11 (https://phytozome.jgi.doe.gov/pz/portal.html) to identify S. viridis SWEET homologs. OsSWEET7a – e were excluded to provide a clearer phylogenetic tree. Protein sequences of Arabidopsis, rice and S. viridis SWEETs were aligned by MUSCLE and iTOL v.3 (http://itol.embl.de/) was used to generate the phylogenetic tree.

**Table S3: Summary of *Oryza sativa* SWEETs.**

| **Name** | **Phytozome Accession number** | **AA length** |
| --- | --- | --- |
| OsSWEET1a | Os01g65880 | 273 |
| OsSWEET1b | Os05g35140 | 261 |
| OsSWEET2a | Os01g36070 | 243 |
| OsSWEET2b | Os01g50460 | 230 |
| OsSWEET3a | Os05g12320 | 246 |
| OsSWEET3b | Os01g12130 | 252 |
| OsSWEET4 | Os02g19820 | 259 |
| OsSWEET5 | Os05g51090 | 237 |
| OsSWEET6a | Os01g42110 | 259 |
| OsSWEET6b | Os01g42090 | 254 |
| OsSWEET7a | Os09g08030 | 206 |
| OsSWEET7b | Os09g08440 | 375 |
| OsSWEET7c | Os12g07860 | 240 |
| OsSWEET7d | Os09g08490 | 62 |
| OsSWEET7e | Os09g08270 | 98 |
| OsSWEET11 | Os08g42350 | 307 |
| OsSWEET12 | Os03g22590 | 300 |
| OsSWEET13 | Os12g29220 | 296 |
| OsSWEET14 | Os11g31190 | 303 |
| OsSWEET15 | Os02g30910 | 319 |
| OsSWEET16 | Os03g22200 | 328 |


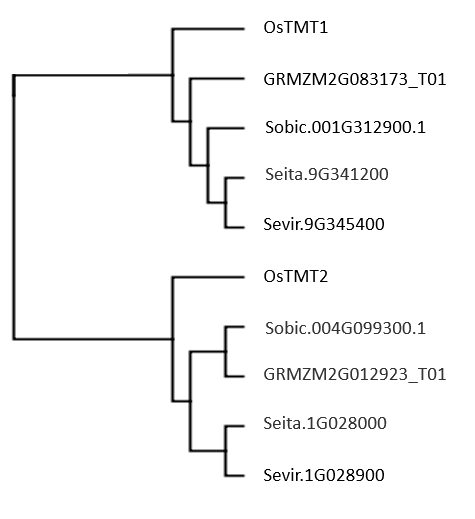


**Figure S4: Phylogenetic analysis of putative *S. viridis* TMT transcripts Sevir.9G345400 and Sevir.1G028900 and protein homologs from related species *Oryza sativa (Os), Zea mays, Sorghum bicolor*, and *Setaria italica.*** Protein sequences of previously characterised OsTMT1 (NCBI Accession No.: GU066765) and OsTMT2 (NCBI Accession No.: GU066766) were used to identify homologs in related grass species using the BLAST algorithm in Phytozome. The phylogenetic tree was produced in Geneious v8.2 (http://www.geneious.com; Kearse et al., 2012).

**Table S4: Significance of Pearson’s correlation coefficients calculated by SPSS**

See attached excel spreadsheet.


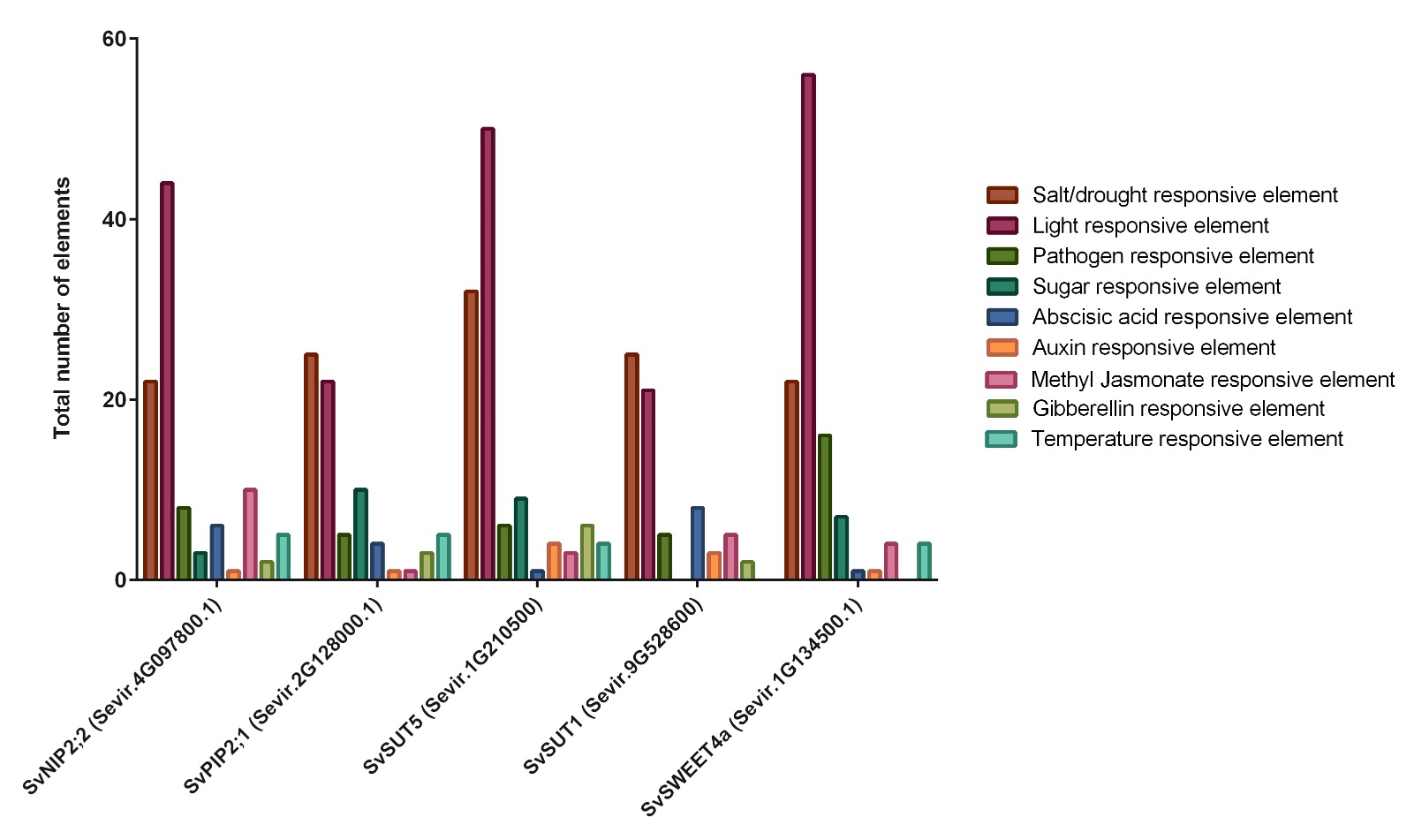


**A**

**
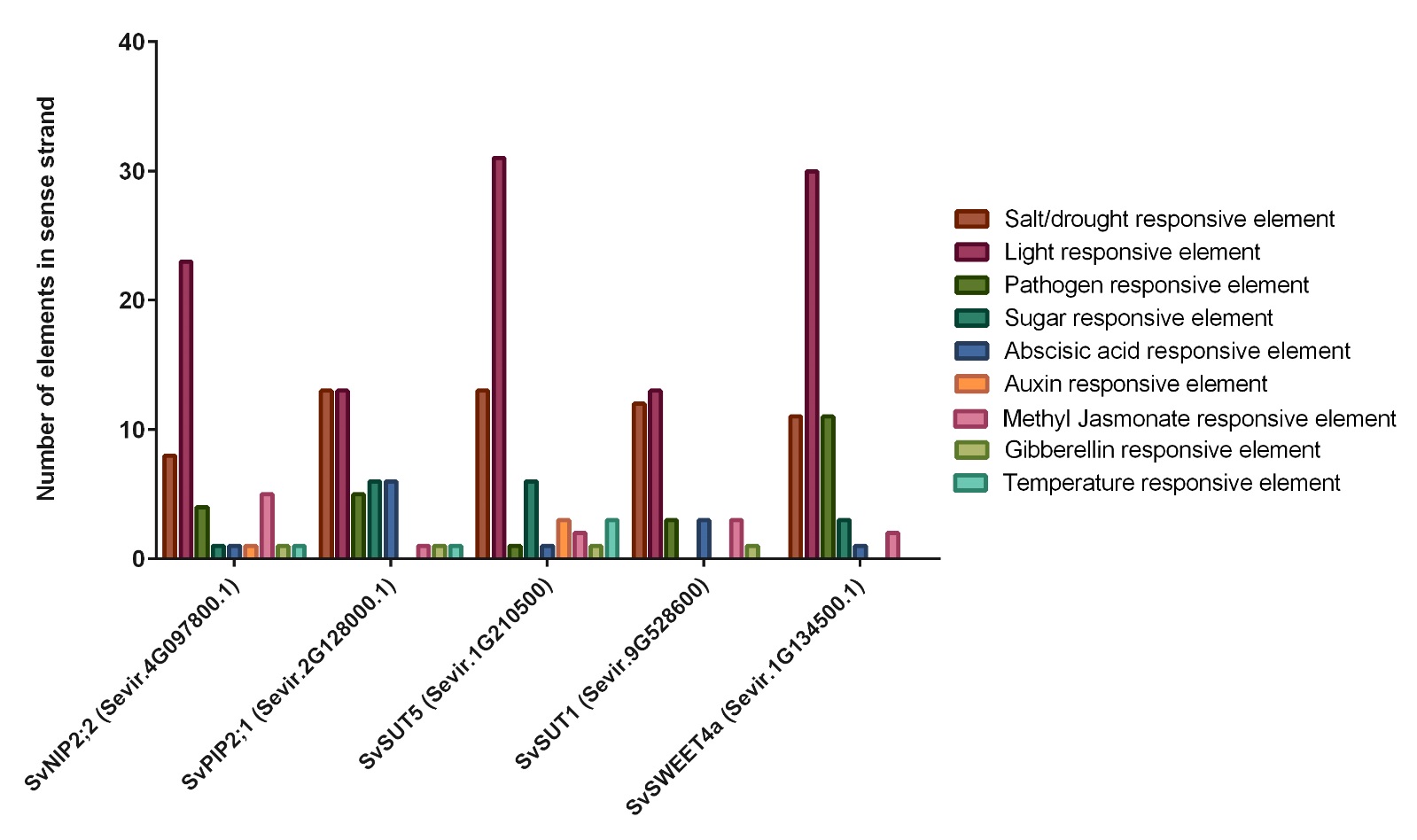
**

**B**

**Figure S5: Summary of *cis-*acting regulatory elements identified in the promoter regions of the aquaporins *SvPIP2;1* and *SvNIP2;2*, and the putative sugar transporter genes *SvSUT1, SvSUT5* and *SvSWEET4a.*** The promoter region 1.5kb upstream of the transcriptional start site of the aquaporins *SvPIP2;1* and *SvNIP2;2*, and the putative sugar transporters *SvSUT1, SvSUT5,* and *SvWEET4a* were searched to identify *cis-*acting regulatory elements registered through the online database PlantCARE (<http://bioinformatics.psb.ugent.be/webtools/plantcare/html/>) and described in Ibraheem et al., (2010). *Cis­­*-acting regulatory elements are grouped by similar function. Salt/drought responsive elements: DRE, MYC, MYB, Erd1, MBS; Light responsive elements: Box4, I-box, GATA, GAP, CATT motif, G-box, MRE, TCCC-motif, MNK1, MNF1, AP1, GAG, ACA motif, AE-box, GA motif, LAMP element; Pathogen responsive elements: W-box, GT-1, GCC-box, TC-rich repeat; Sugar responsive elements: Pyrimidine box, sucrose box, A-box; Abscisic acid responsive elements: ABRE, AuxRR-core, Motif Iib; Auxin responsive elements: TGA element, ARF; Methyl Jasmonate responsive elements: MeJA motif; Gibberellin responsive elements: P-box, TATC-box, GARE motif; Temperature responsive element: LTRE, HSE. **(A)** Sum of sense and anti-sense strand ­*cis*-acting regulatory elements. **(B)** Sense strand *cis-*regulatory elements.

**
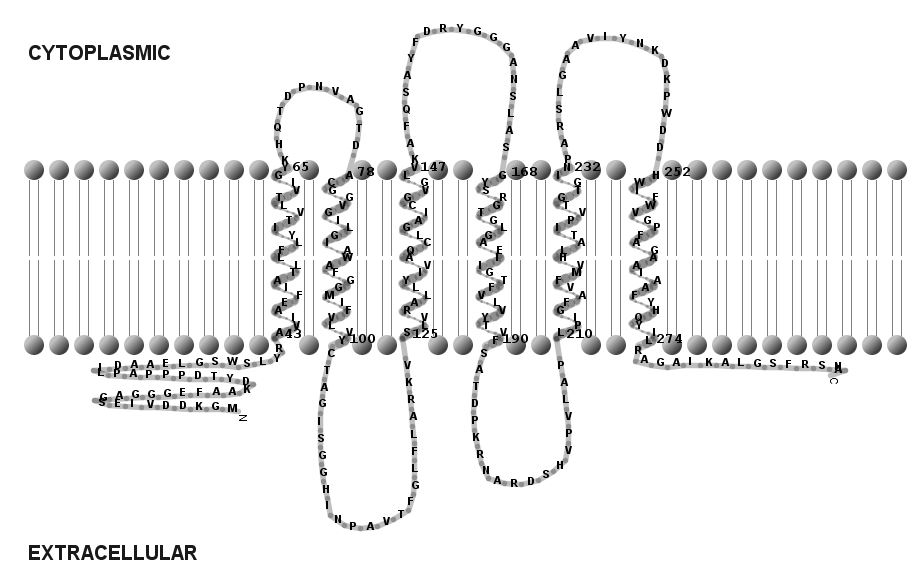
**

**(B)**

**(A)**


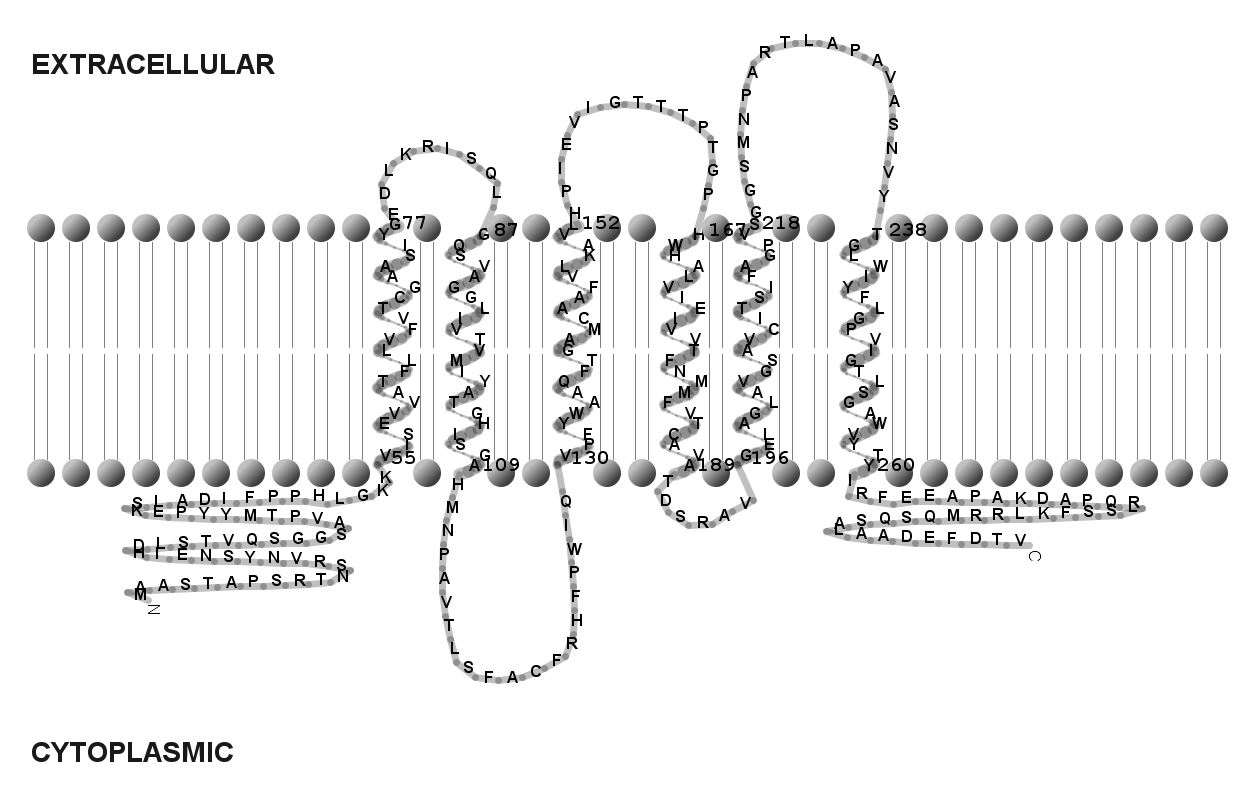


**Figure S5: Topological model of the predicted transmembrane structure of *S. viridis* aquaporins SvPIP2;1 and SvNIP2;2. (A)** SvPIP2;1 **(B)** SvNIP2;2. Topological predictions of candidate genes were generated by TMHMM (Krogh et al., 2001) and show transmembrane helices (1-6) and intra- and extracellular connecting loops (A-E).
